# Supplementary material for: Screens in aging-relevant human ALS-motor neurons identify MAP4Ks as therapeutic targets for the disease
Source: Cell Death Dis. 2024 Jan 4;15(1):4. doi: 10.1038/s41419-023-06395-7 (PMC10766628; doi:10.1038/s41419-023-06395-7)
Supplement: Supplementary file 1 — Supplementary Figures S1-8 [file 41419_2023_6395_MOESM1_ESM.pdf]

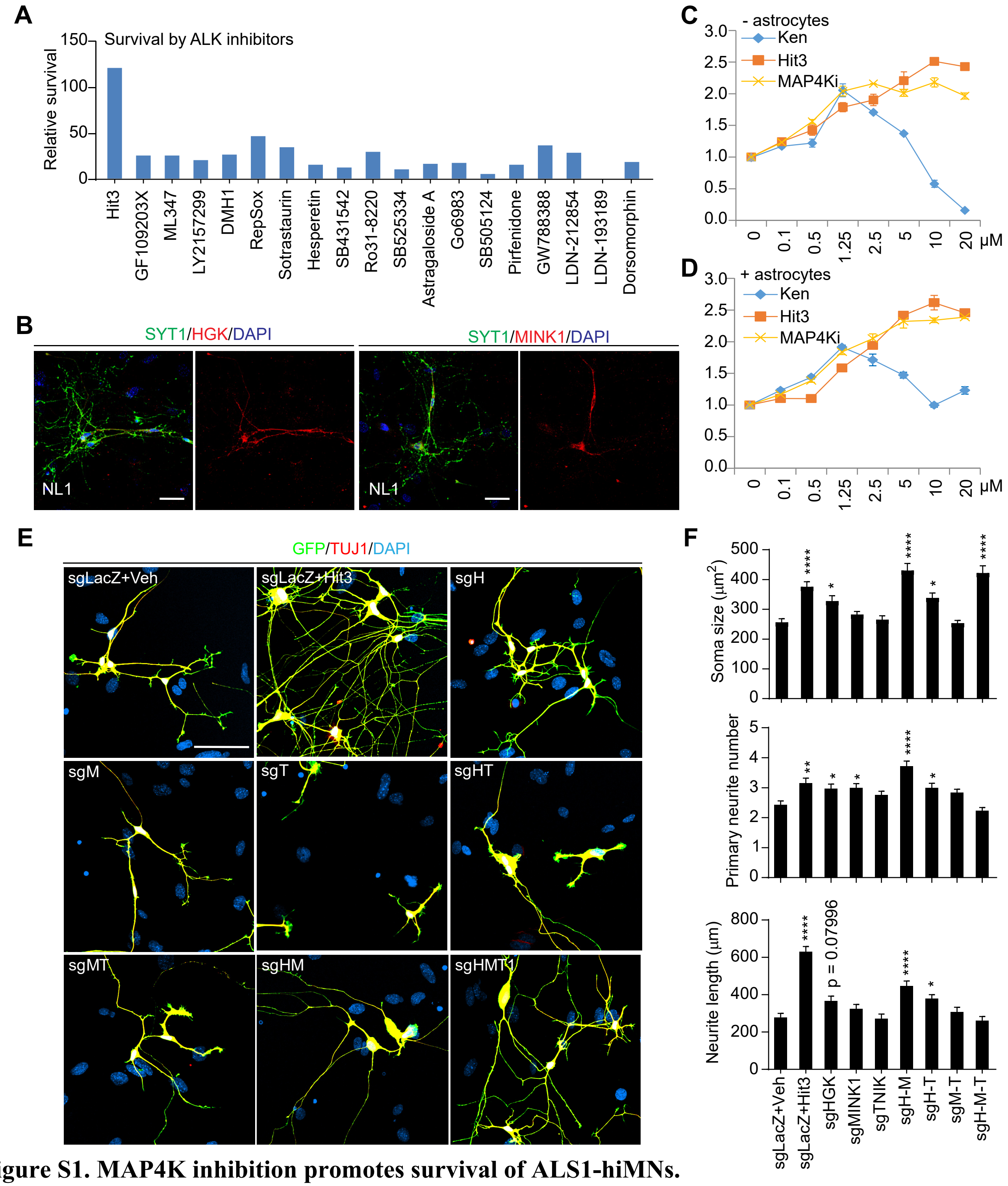

Figure S1. MAP4K inhibition promotes survival of ALS1-hiMNs.
A. A lack of survival effect on ALS1-hiMNs by ALK inhibitors at 3 days post replating.
B. HGK and MINK1 expression in NL1-hiMNs at 52 dpi. SYT1, Synaptotagmin 1. Scale bars, 100 μm.
C, D. Dose-dependent effect of the indicated chemicals on survival of ALS1-hiMNs with or without cocultured astrocytes at 1 week post replating (mean ± SEM; n = 4 independent samples at each concentration). Ken, kenpaullone; MAP4Ki, PF06260933.
E. Morphological changes of ALS1-hiMNs under the indicated conditions at 3 weeks post replating on astrocytes. Genes were downregulated via sgRNAs and CRISPR-Cas9. H, HGK; M, MINK1; T, TNIK. Scale bar, 100 μm.
F. Quantification of soma size, primary neurite number and length (mean ± SEM; n = 50 neurons per group for soma size; n = 100 neurons per group for neurite number and length; *$p < 0.05$, **$p < 0.01$, and ****$p < 0.0001$, one-way ANOVA, when compared to control sgLacZ+Veh group).

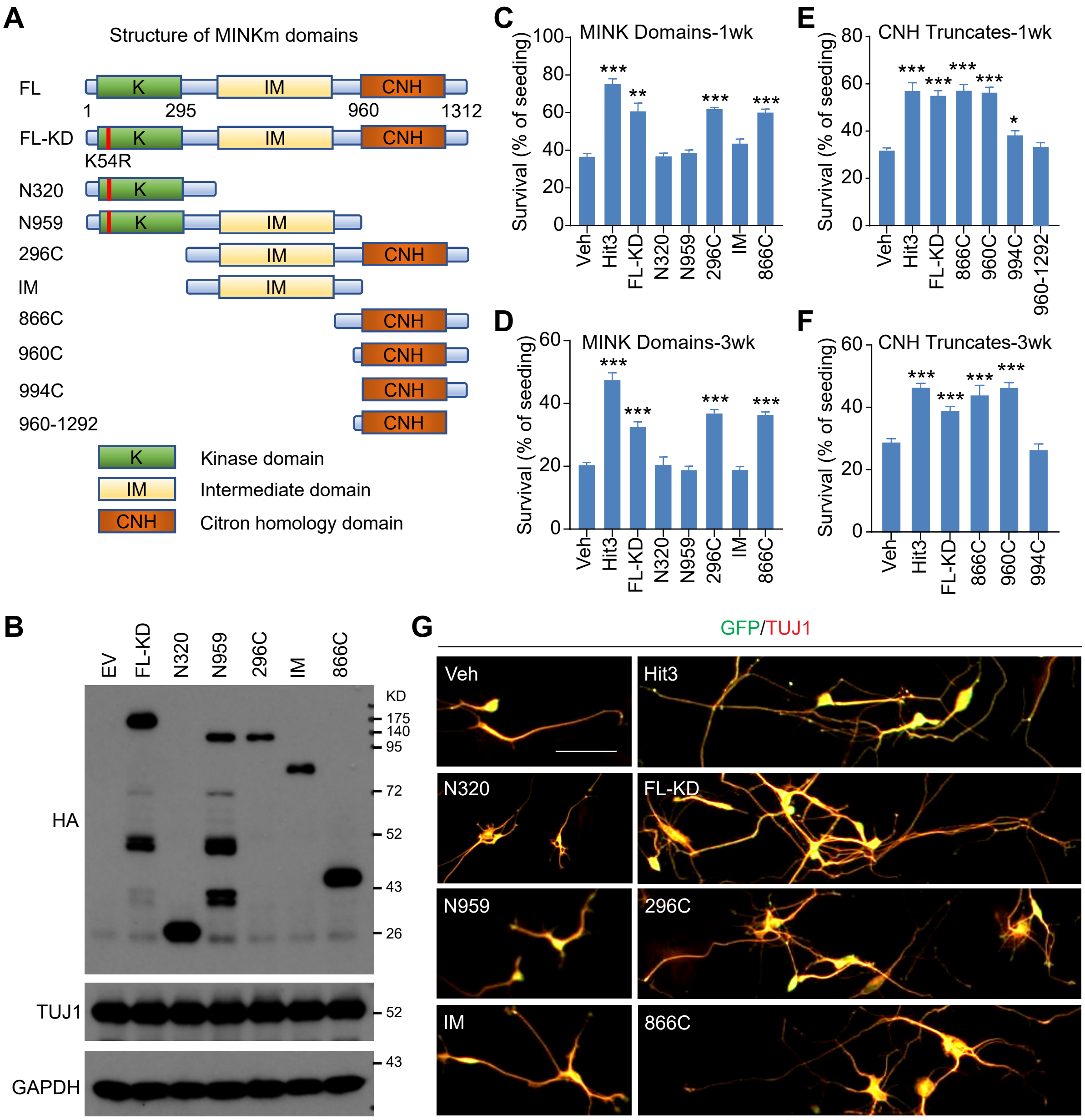

Figure S2. CNH domain of MINK1 improves survival of ALS1-hiMNs.

A. Schematic diagrams of MINK1 protein and its truncations. The point mutation, K54R, renders MINK1 inactive.

B. Western blotting analysis of ectopic HA-tagged proteins in ALS1-hiMNs purified at 14 dpi.

C, D. The CNH-containing domain (866C: aa866-1312) is sufficient to improve survival of ALS1-hiMNs at the indicated time point (mean ± SEM; n = 4; **$p < 0.01$ and ***$p < 0.001$ when compared to the Veh-treated samples, Student's t-test).

E, F. The CNH domain (960C: aa960-1312) mimics Hit3's effect on survival of ALS1-hiMNs at the indicated time point (mean ± SEM; n = 4; *$p < 0.05$ and ***$p < 0.001$ when compared to the Veh-treated samples, Student's t-test).

G. The kinase-dead MINK mutant and its functional domains recapitulated the effect of Hit3 on morphology of ALS1-hiMNs 1-week post replating. Scale bar, 100 µm.

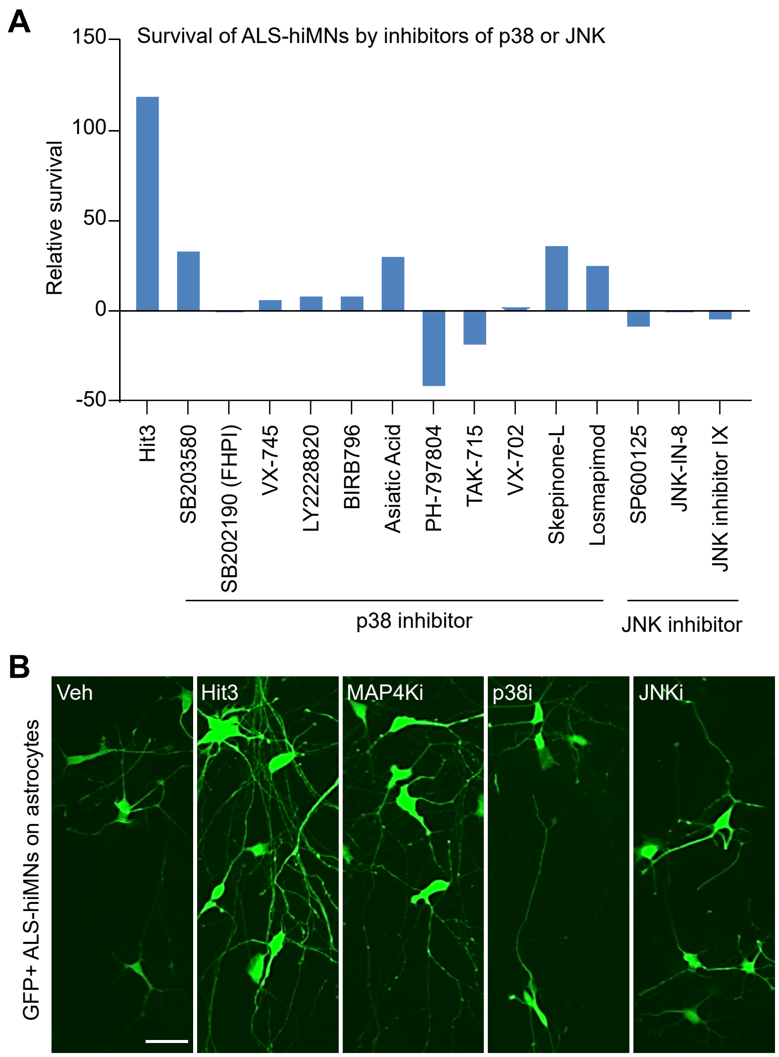

Figure S3. A lack of protective effect of p38 or JNK inhibitors on ALS1-hiMNs.

A. Survival assays of ALS1-hiMNs treated with the indicated inhibitors at 3 days post replating. Data were from the primary screens.

B. Effect of the selected chemicals on morphology of ALS1-hiMNs at 1 week post replating on astrocytes. MAP4Ki, PF06260933; p38i, p38 inhibitor SB203580; JNKi, JNK inhibitor SP600125.

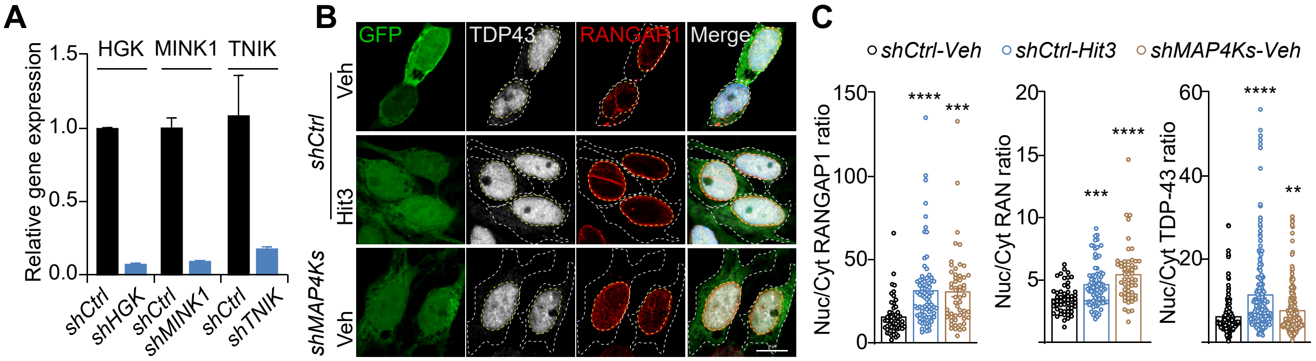

Figure S4. Downregulation of MAP4Ks, resembling Hit3 treatments, promotes nuclear localization of key proteins in ALS1-hiMNs.

A. qRT-PCR analysis of shRNA-mediated knockdowns in human fibroblasts at 5 dpi (mean ± SEM; n = 3).

B. Confocal images of RANGAP1 and TDP-43 in ALS1-hiMNs cocultured with astrocytes at 51 dpi. The soma and nucleus are outlined. Scale bar, 10 µm.

C. Downregulation of MAP4Ks, like Hit3 treatments, improves nuclear localization of the indicated proteins in ALS1-hiMNs at 51 dpi (mean ± SEM; RANGAP1 and RAN: n = 54, 79 and 55 for the indicated groups, respectively; TDP-43: n = 198, 212 and 233 for the indicated groups, respectively; $^{**}p < 0.01$, $^{***}p < 0.001$, and $^{****}p < 0.0001$, one-way ANOVA).

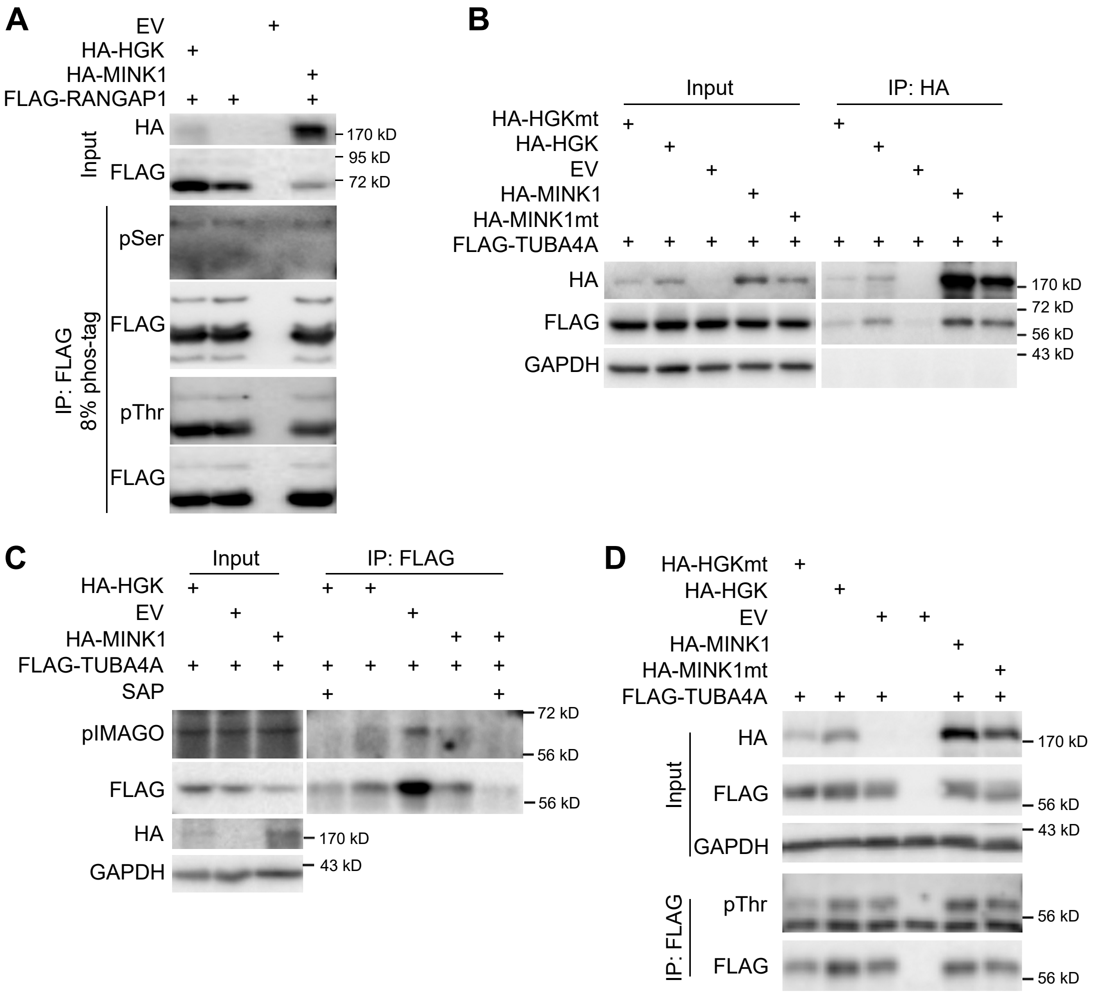

Figure S5. Neither RANGAP1 nor TUBA4A is phosphorylated by MAP4Ks.

A. Phos-tag SDS-PAGE and western blots failed to detect phosphorylation of RANGAP1 by MAP4Ks.

B. Co-IP results showing interactions between TUBA4A and HGK or MINK1 or their kinase-dead mutants.

C, D. pIMAGO kit or pThr phospho-antibody failed to detect phosphorylation of TUBA4A by HGK or MINK1.

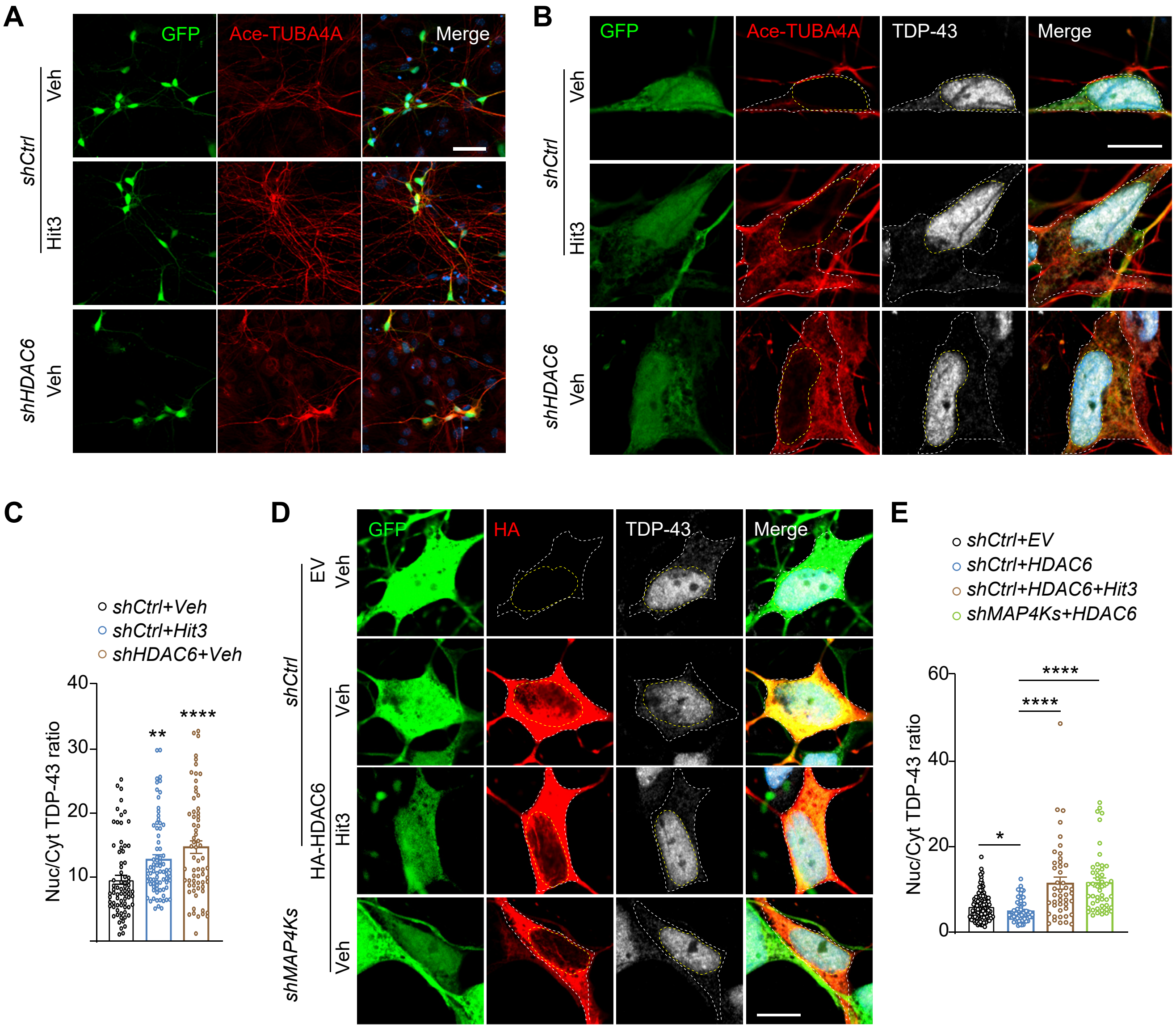

Figure S6. HDAC6 regulates TUBA4A acetylation and TDP-43 subcellular distribution.

A. Confocal images of ac-TUBA4A in ALS1-hiMNs cocultured with astrocytes at 28 dpi. Scale bar, 50 µm.

B. Confocal images of ac-TUBA4A and TDP-43 in ALS1-hiMNs cocultured with astrocytes at 28 dpi. Scale bar, 10 µm.

C. HDAC6 knockdown, like Hit3 treatments, improves nuclear localization of TDP-43 in ALS1-hiMNs (mean ± SEM; n = 73, 78 and 66 for the indicated groups, respectively; $^{**}p < 0.01$ and $^{****}p < 0.0001$, one-way ANOVA).

D. Confocal images of TDP-43 distribution in ALS1-hiMNs cocultured with astrocytes at 28 dpi. Scale bar, 10 µm.

E. Inhibition or knockdown of MAP4Ks reverses HDAC6's effect on the subcellular distribution of TDP-43 in ALS1-hiMNs (mean ± SEM; n = 103, 67, 45 and 48 for the indicated groups, respectively; $^{*}p < 0.05$ and $^{****}p < 0.0001$, one-way ANOVA).

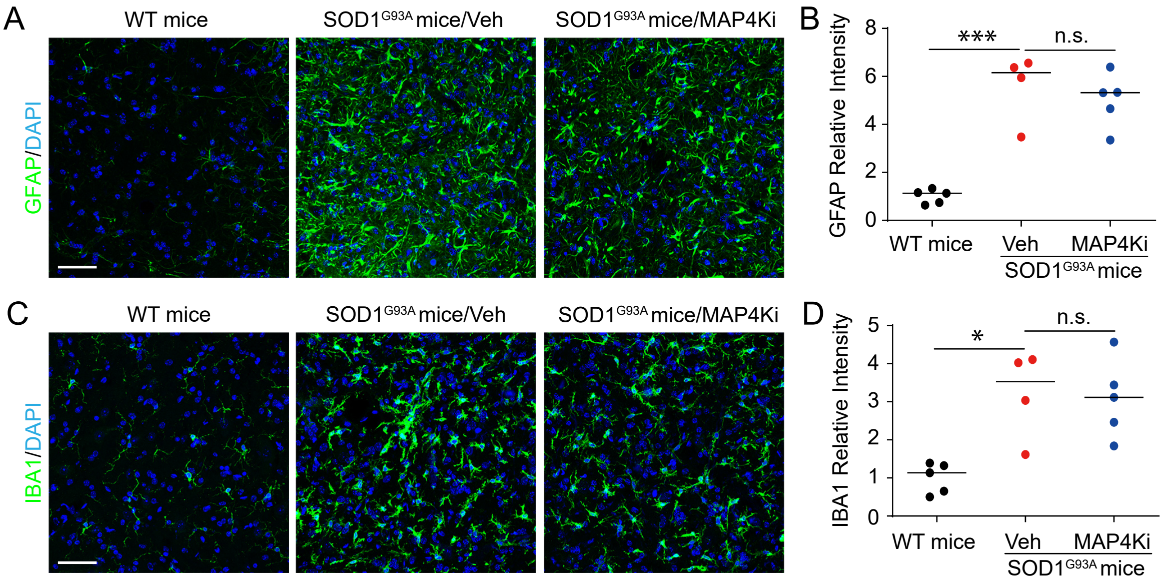

Figure S7. MAP4Ki has no effect on reactive gliosis in SOD1^{G93A} mice.

A. Confocal images showing GFAP expression in the ventral horn of the gray matter of the lumbar spinal cord. Scale bar, 50 µm.

B. Quantification of GFAP relative intensity (mean ± SEM; n = 5, 4, and 5 for the indicated groups, respectively; female mice; ***$p < 0.001$; n.s., not significant; one-way ANOVA).

C. Confocal images showing IBA1 expression in the ventral horn of the gray matter of the lumbar spinal cord. Scale bar, 50 µm.

D. Quantification of IBA1 relative intensity (mean ± SEM; n = 5, 4, and 5 for the indicated groups, respectively; female mice; *$p < 0.05$; n.s., not significant).

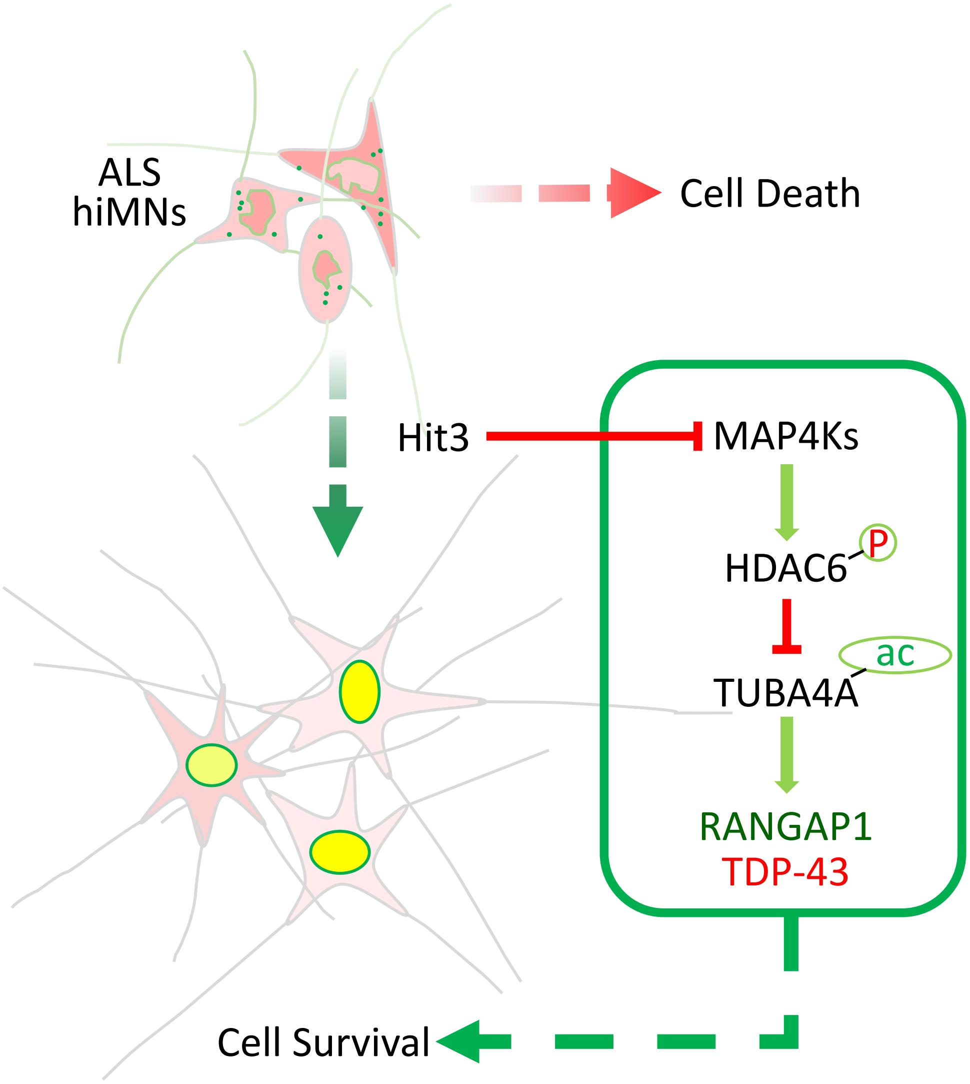

Figures S8. A schematic showing how Hit3 improves survival of ALS-hiMNs.
hiMNs derived from ALS patient' skin fibroblasts degenerate with time in culture. Such degeneration could be rescued by the small molecule Hit3. Mechanistically, Hit3 functions as an inhibitor of MAP4Ks to block phosphorylation of HDAC6, resulting in enhanced acetylation of TUBA4A, stabilized microtubules, and nuclear localization of RANGAP1 and TDP-43. ALS, amyotrophic lateral sclerosis; HDAC6, histone deacetylase 6; hiMNs, human-induced motor neurons directly converted from adult skin fibroblasts; MAP4Ks, MAP kinase kinase kinase kinases; RANGAP1, Ran GTPase-activating protein 1; TDP-43, TAR DNA-binding protein 43; TUBA4A, tubulin alpha-4A.
