# Supplementary material for: Screens in aging-relevant human ALS-motor neurons identify MAP4Ks as therapeutic targets for the disease
Source: Cell Death Dis. 2024 Jan 4;15(1):4. doi: 10.1038/s41419-023-06395-7 (PMC10766628; doi:10.1038/s41419-023-06395-7)
Supplement: Supplementary file 2 — Table S1 [file 41419_2023_6395_MOESM2_ESM.pdf]

Table S1. Sources of fibroblasts examined in this work.

Name in this study	Alternative Name	Company (Catalog ID)	Origin	Age	Gender	Disease
NL1	AG05811	Coriell	Skin	71 Years	Female	Apparently Healthy
NL2	AG08517	Coriell	Skin	66 Years	Female	Apparently Healthy
NL3	AG09969	Coriell	Skin	53 Years	Male	Apparently Healthy
NL4	AG11733	Coriell	Skin	70 Years	Female	Apparently Healthy
NL5	AG12989	Coriell	Skin	47 Years	Male	Apparently Healthy
ALS1	ND29563	Coriell	Skin	37 Years	Male	FUS: R522R
ALS2	ND39027	Coriell	Skin	50 Years	Female	FUS: H517Q
ALS3	ND40077	Coriell	Skin	47 Years	Female	FUS: R521G
ALS4	ND29774	Coriell	Skin	68 Years	Female	SOD1: D90A
ALS5	F09229	Baloh Lab	Skin	46 Years	Male	C9orf72 hexanucleotide repeat expansion (6-8kb expanded allele)
ALS6	ND32947	Coriell	Skin	64 Years	Male	TDP43: G298S
ALS7	C9-4	Corey Lab	Skin	61 Years	Male	C9orf72 hexanucleotide repeat expansion, Bulbar
ALS8	C9-5	Corey Lab	Skin	69 Years	Female	C9orf72 hexanucleotide repeat expansion, Bulbar, FTD suspected
