# Supplementary material for: Screens in aging-relevant human ALS-motor neurons identify MAP4Ks as therapeutic targets for the disease
Source: Cell Death Dis. 2024 Jan 4;15(1):4. doi: 10.1038/s41419-023-06395-7 (PMC10766628; doi:10.1038/s41419-023-06395-7)
Supplement: Supplementary file 4 — Table S3 [file 41419_2023_6395_MOESM4_ESM.pdf]

Table S3. Chemical information of Top 15 hits and controls.

No.	Plate No.	CAT No.	Product Name	Secondary Hit Score	Targets
Hit1	L1700-20c3	**S7193**	**1-Azakenpaullone**	132.04	GSK-3
Hit2	L1700-2a3	**S1134**	**AT9283**	124.97	JAK,Aurora Kinase,Bcr-Abl
Hit3	L1700-21a2	**S7359**	**K02288**	120.10	TGF-beta/Smad
Hit4	L1700-19g11	**S7145**	**AZD1080**	119.17	GSK-3
Hit5	L1700-20e6	**S7253**	**AZD2858**	116.00	GSK-3
Hit6	L1700-1b8	**S1075**	**SB216763**	111.80	GSK-3
Hit7	L1700-1d6	**S1055**	**Enzastaurin (LY317615)**	109.33	PKC
Hit8	L1700-3d5	**S1274**	**BX-795**	97.62	IκB/IKK,PDK-1
Hit9	L1700-14c11	**S2924**	**CHIR-99021 (CT99021) HCl**	89.27	GSK-3
Hit10	L1700-9h4	**S2201**	**BMS-794833**	87.41	VEGFR,c-Met
Hit11	L1700-14g3	**S2843**	**BI-D1870**	60.28	S6 Kinase
Hit12	L1700-5e5	**S1570**	**KU-60019**	57.73	ATM/ATR
Hit13	L1700-20f3	**S7198**	**BIO**	55.40	GSK-3
Hit14	L1700-13h5	**S2735**	**MK-8776 (SCH 900776)**	49.07	CDK/Chk
Hit15	L1700-20a5	**S7223**	**RepSox**	46.27	TGF-β
A83			**A83-01**		TGF-β
AC	Hit3 analog		**LDN214117**		TGF-β
ALKi1			**LDN-193189**		TGF-β
ALKi2	L1700-20a1	**S7147**	**LDN-212854**		TGF-β
PC	Positive control		**Kenpaullone**	100.00	GSK-3/HGK
NC	Negative control		**DMSO**		
