# Supplementary material for: Screens in aging-relevant human ALS-motor neurons identify MAP4Ks as therapeutic targets for the disease
Source: Cell Death Dis. 2024 Jan 4;15(1):4. doi: 10.1038/s41419-023-06395-7 (PMC10766628; doi:10.1038/s41419-023-06395-7)
Supplement: Supplementary file 6 — Table S5 [file 41419_2023_6395_MOESM6_ESM.pdf]

Hit3 (K02288)

	Plasma		Brain		Spinal cord	
Time (min)	Conc (ng/ml)	std. dev.	Conc (ng/g)	std. dev.	Conc (ng/g)	std. dev.
10	165	52.5	178	29.4	213	30.3
30	29.0	10.4	11.7	8.61	16.7	6.17
90	61.8	5.54	2.14	3.71	6.55	4.67
180	145	31.2	0	0	2.82	2.47
360	132	28.6	17.0	29.4	1.47	2.54
960	15.2	0.65	0	0	0	0
1440	3.09	5.13	0	0	0	0

MAP4Ki (PF-6260933)

	Plasma		Spinal Cord		Brain	
Time (min)	Conc (ng/ml)	std. dev.	Conc (ng/g)	std. dev.	Conc (ng/g)	std. dev.
10	2195	460	3792	788	5234	198
30	1435	375	4209	388	3827	116
120	348	57	867	263	885	9
240	106	50	316	12	317	128
480	94	19	200	11	247	97

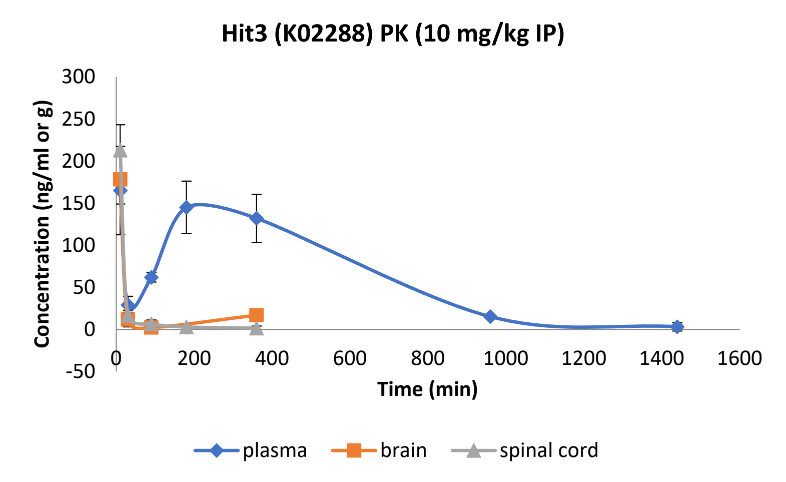

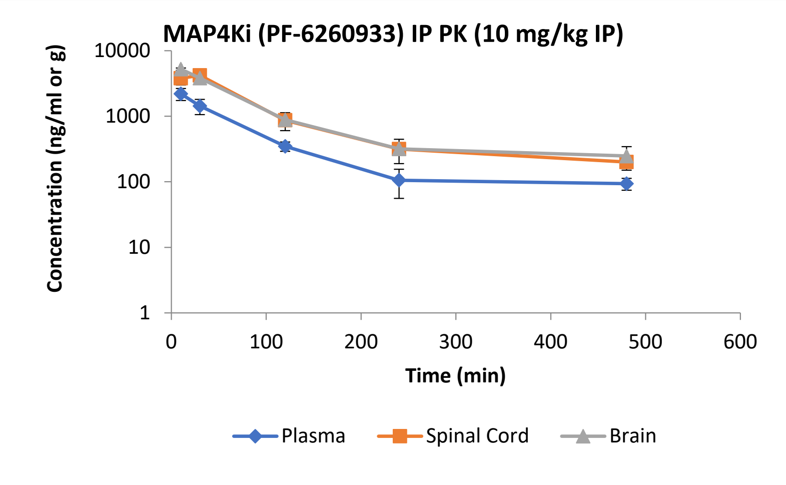

*Brain concentrations were corrected for known volume of blood in brain but no correction value is known for spinal cord so that value is uncorrected.
