# Supplementary material for: Screens in aging-relevant human ALS-motor neurons identify MAP4Ks as therapeutic targets for the disease
Source: Cell Death Dis. 2024 Jan 4;15(1):4. doi: 10.1038/s41419-023-06395-7 (PMC10766628; doi:10.1038/s41419-023-06395-7)
Supplement: Supplementary file 10 — Figures S9 [file 41419_2023_6395_MOESM10_ESM.pdf]

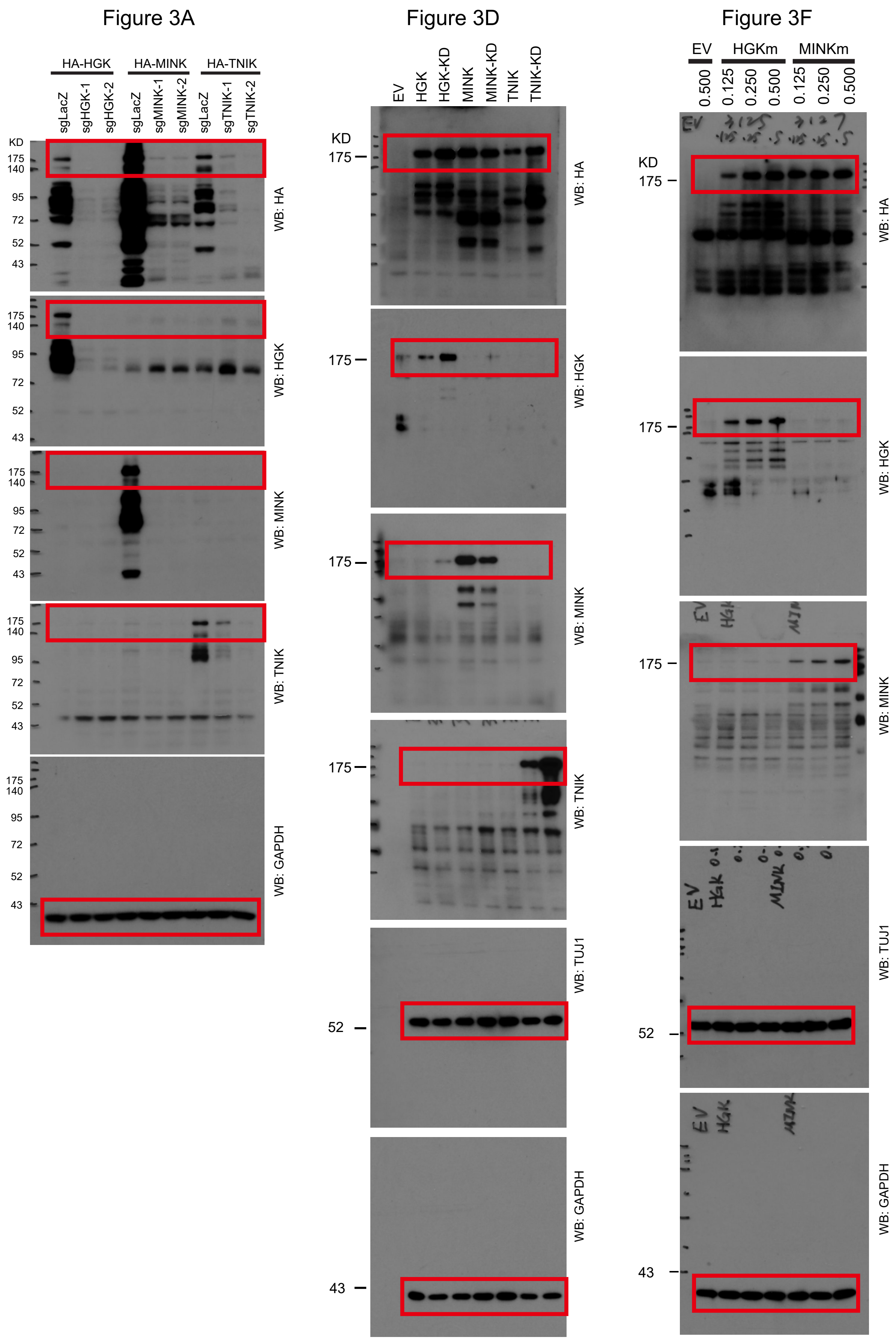

Figure S9-1. Full and uncropped western blots, related to Figure 3.

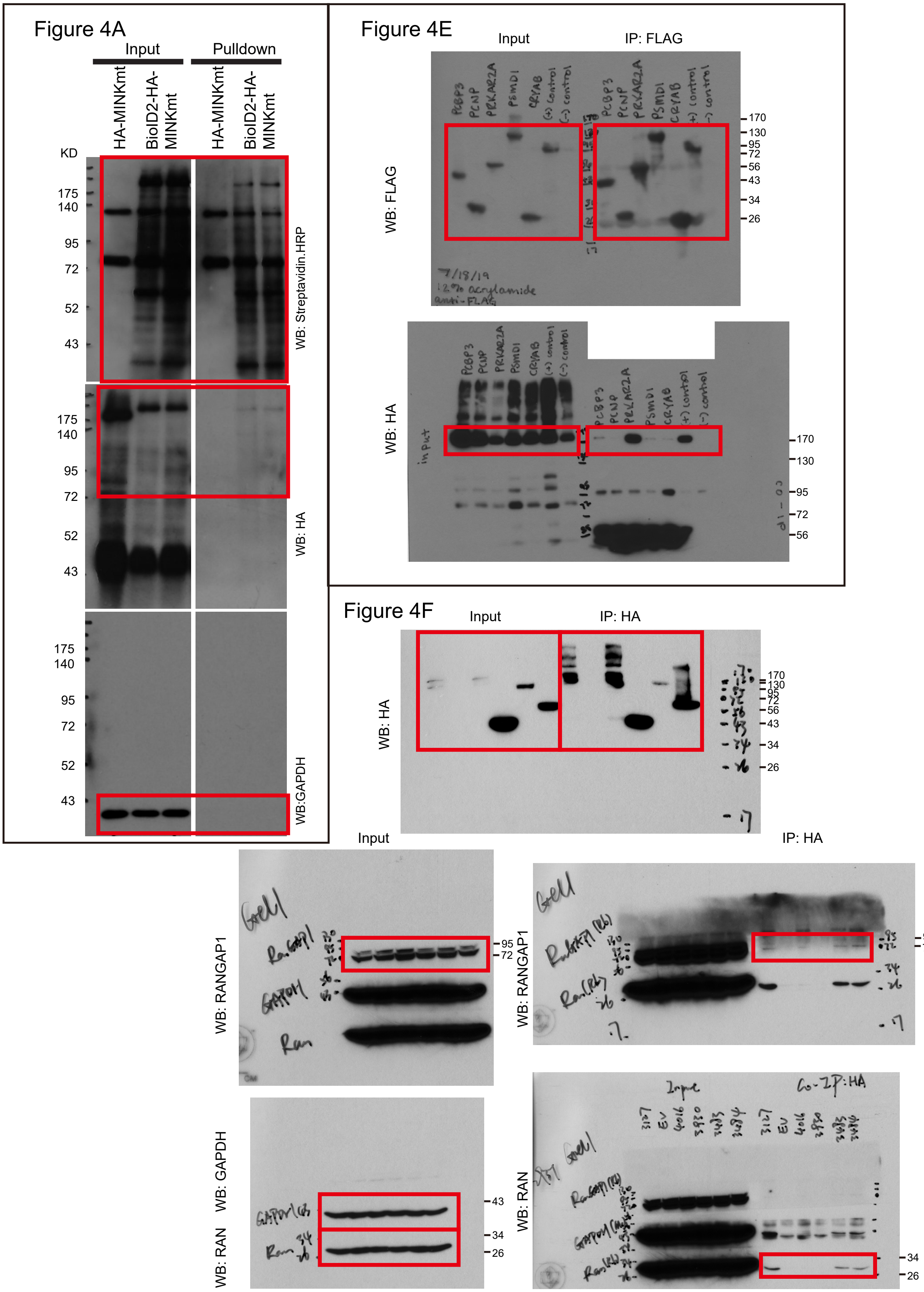

Figure S9-2. Full and uncropped western blots, related to Figure 4.

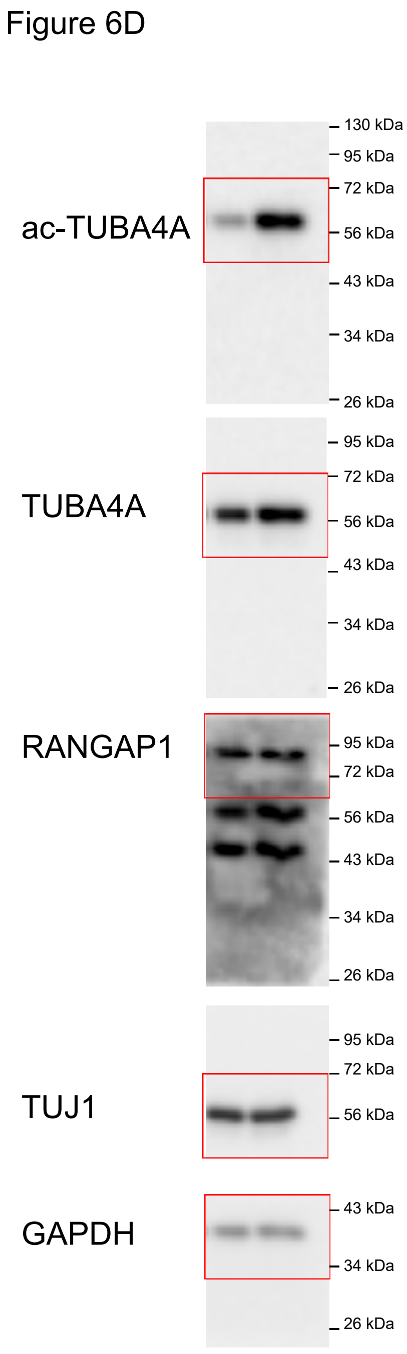

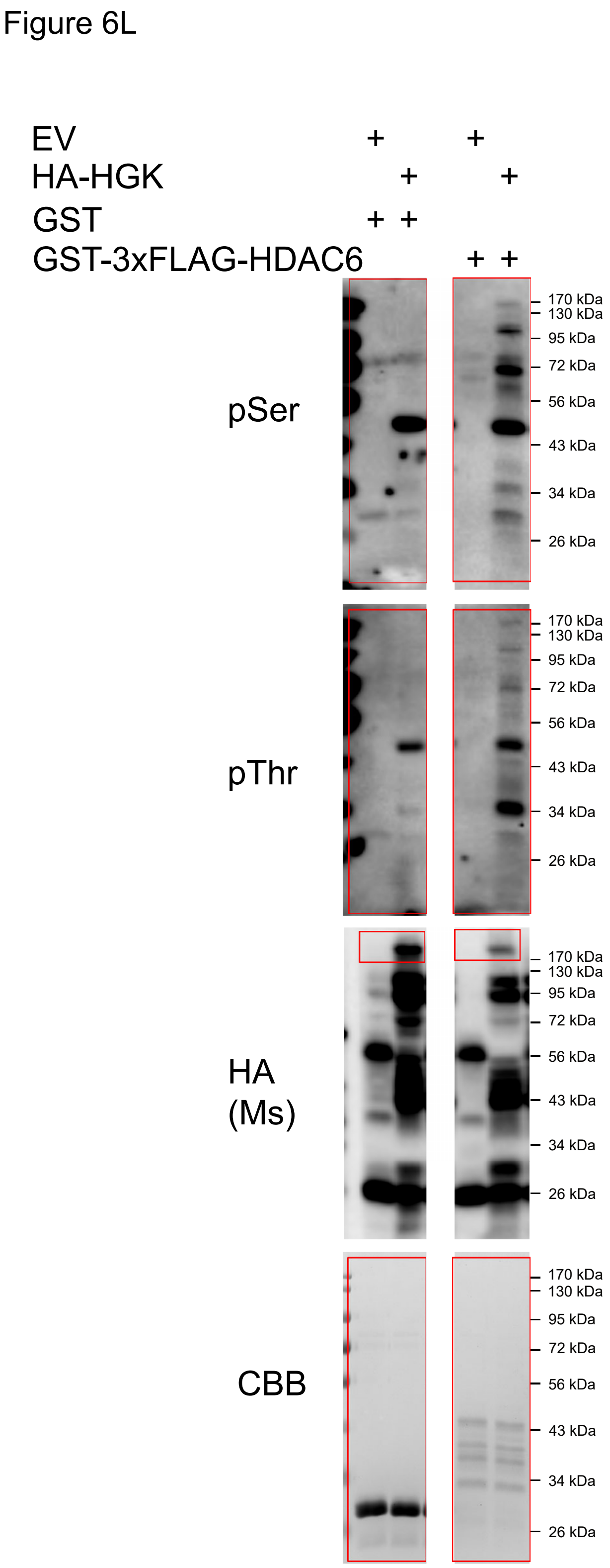

Figure S9-3. Full and uncropped western blots, related to Figure 6.

Figure S2B

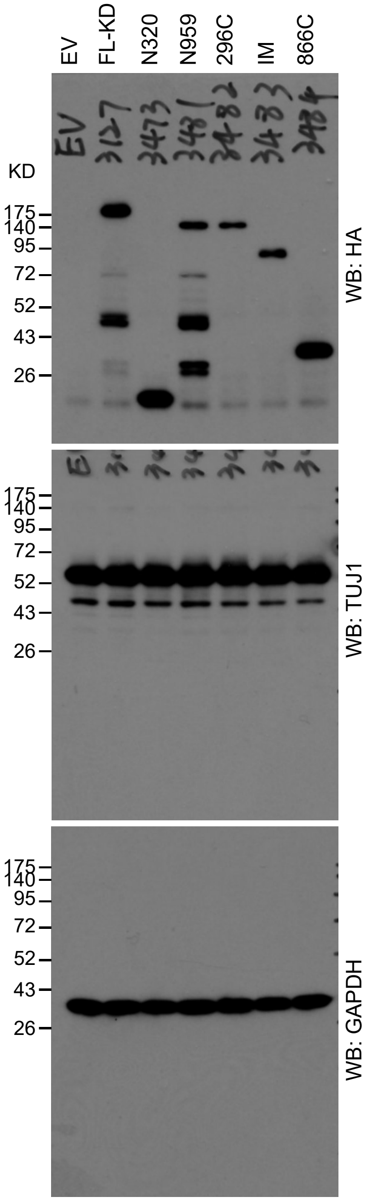

Figure S9-4. Full and uncropped western blots, related to Figure S2.

Figure S5A

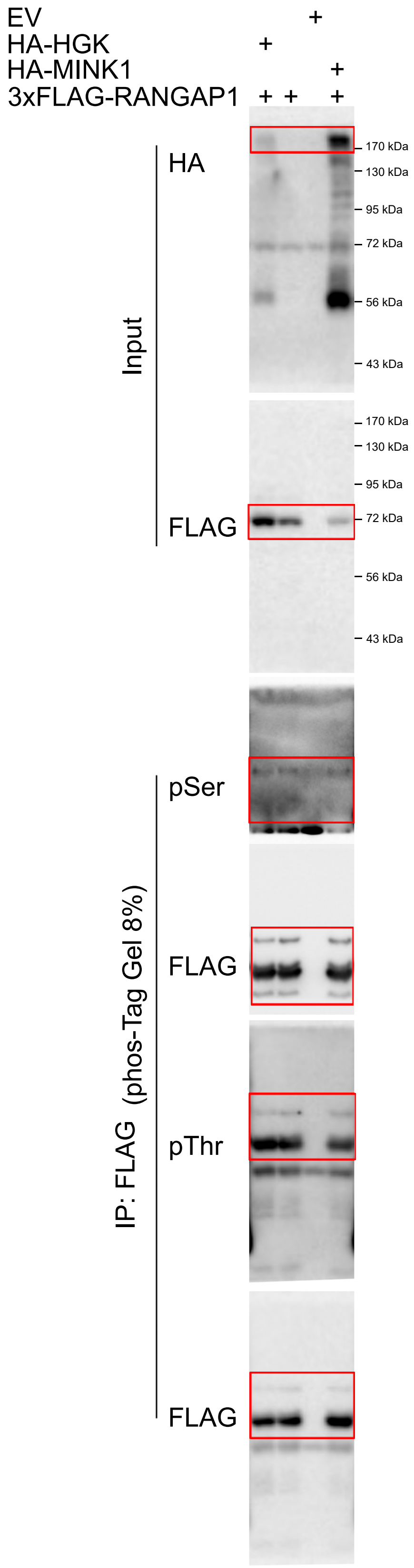

Figure S5B

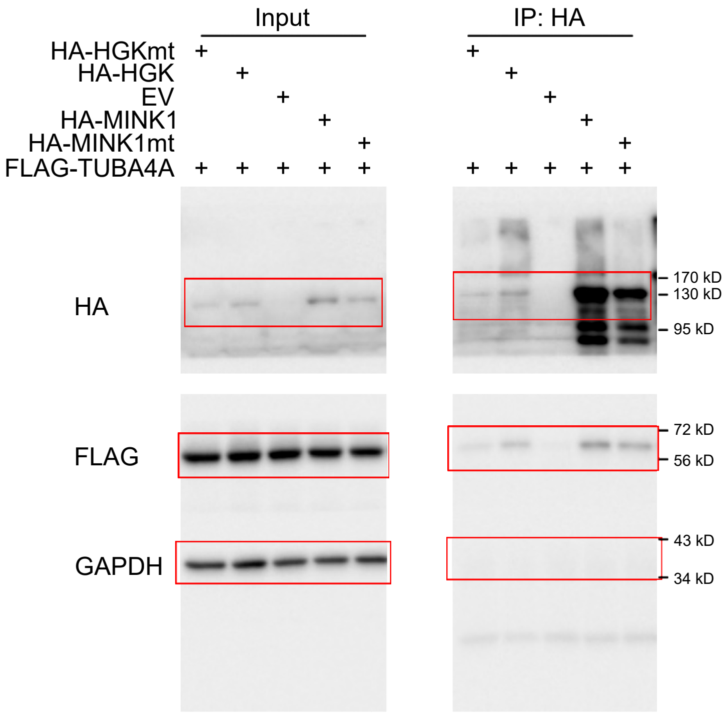

Figure S9-5. Full and uncropped western blots, related to Figure S5A-B.

Figure S5C

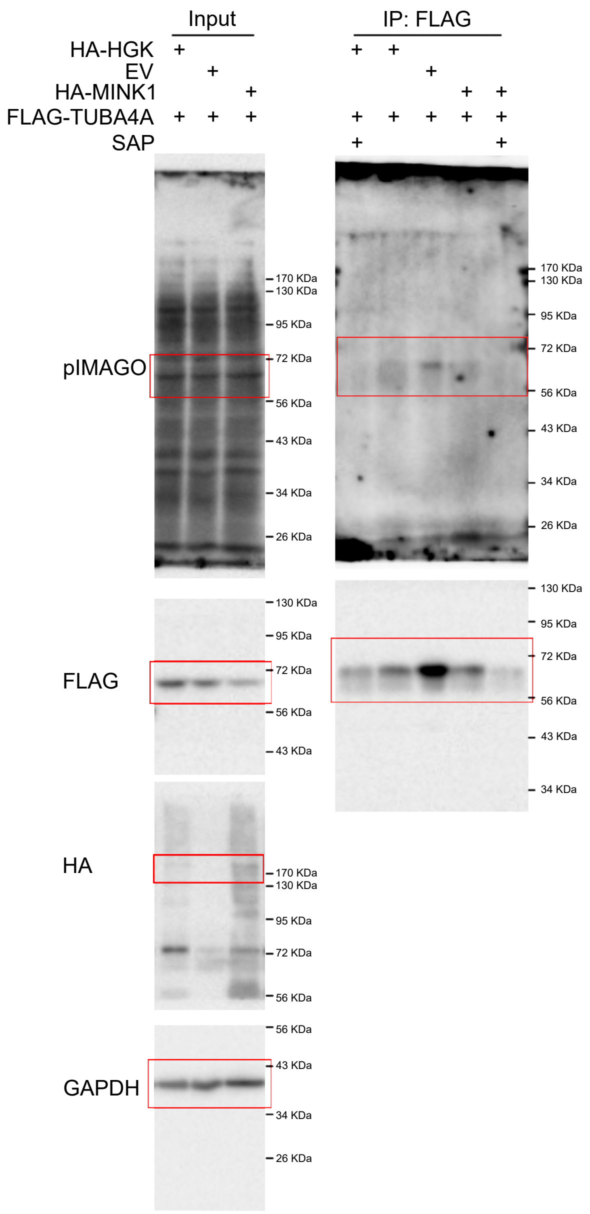

Figure S5D

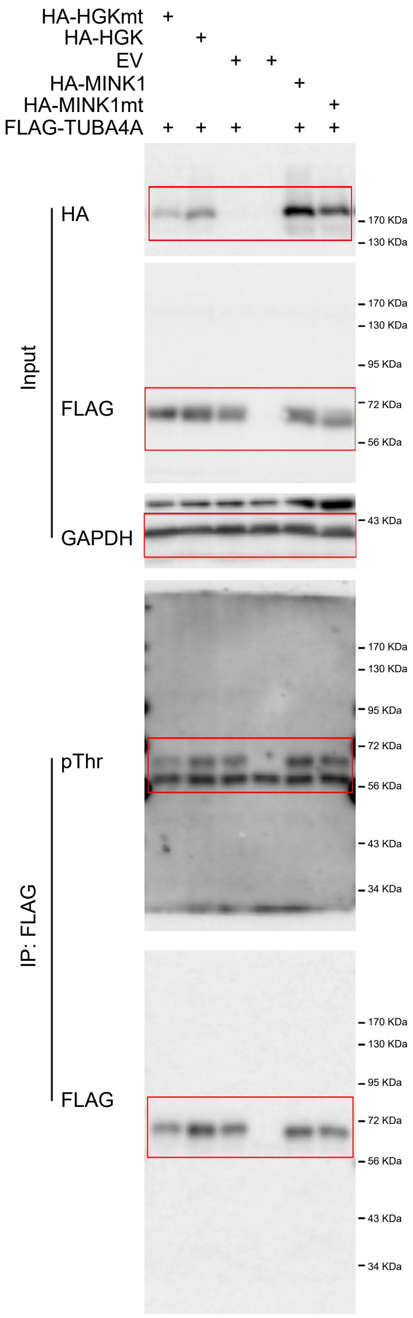

Figure S9-6. Full and uncropped western blots, related to Figure S5C-D.
